# Assessing the influence of music on wine perception among wine professionals

**DOI:** 10.1002/fsn3.554

**Published:** 2017-12-01

**Authors:** Qian (Janice) Wang, Charles Spence

**Affiliations:** ^1^ Crossmodal Research Laboratory Department of Experimental Psychology Oxford University Oxford UK

**Keywords:** crossmodal correspondences, music, wine, wine expertise

## Abstract

Several recent studies have demonstrated that music can significantly influence the eating/drinking experience. It is not clear, however, whether this influence would be moderated by the expertise of the taster. In the experiments reported here, we tested a large group (*N* = 154) of very experienced wine tasters—the majority of whom were professionals working in the wine business—at a winemaking conference. The first study assessed the impact of putatively “sweet” and “sour” soundtracks on taste evaluation, whereas the second study assessed more subtle wine‐specific terminology such as length, balance, and body. The results revealed that the effect of music on wine perception can indeed be demonstrated in wine experts. Moreover, the amount of wine tasting experience, as measured in years, did not moderate the influence of music on sensory and hedonic wine evaluation. This result suggests that the aforementioned auditory modulation of drinking experience is not influenced by the increased analytical abilities afforded by traditional wine tasting expertise.

## 1 INTRODUCTION

Over the last decade, a growing number of studies have started to address the question of whether what we hear can influence what we taste (see Knöferle & Spence, 2012; and Spence, 2012; for reviews). One of the major points of interest that has emerged from this body of research is the relationship between music and wine (North, 2012; Spence, 2011; Spence et al., 2013; 2014; Wang & Spence, 2015; see Spence & Wang, 2015a, 2015b, 2015c; for reviews). Indeed, away from the rigors of the science laboratory, wine writers have long been tempted to compare specific wines to particular pieces of music. Take, for example, the following excerpt as a representative example: “Benny Goodman is a Riesling from Joseph Phelps, Louis Martini's wines have the charm and good manners of Glenn Miller. Joe Heitz, though, is surely Armstrong at the Sunset Café; virtuoso, perverse and glorious.” (Johnson, 2005, p. 253).

Recent studies have demonstrated that people do indeed consistently match certain wines with specific pieces of music under forced choice conditions (Spence et al., 2013; Wang & Spence, 2015; see Spence & Wang, 2015a; for a review). For instance, Spence et al. (2013) demonstrated that Domaine Didier Dagueneau Pouilly Fumé, a crisp white wine, matched Mozart's Flute Quartet in D major—Movement 1 significantly better than Tchaikovsky's String Quartet No 1—Movement 2. The reverse pattern of results—that is, a better match with Tchaikovsky than Mozart—was observed when participants tasted a glass of Chateau Margaux, a rich red wine instead. Furthermore, in a subsequent experiment, tasting the wines while listening to matching music resulted in a small but significant increase in people's rated enjoyment of the wine‐drinking experience as compared to tasting the same wines in silence.

Moreover, music also alters other aspects of participants’ taste experiences beyond liking (see Spence & Wang, 2015b; for a review). For instance, at a live classical music performance where both a New Zealand Sauvignon Blanc and an Argentine Malbec were served, both wines were reported as tasting more acidic when Debussy's Jardin sous la Pluie, a fast, high‐pitched piano piece, was played. In contrast, both wines were rated as tasting more fruity, whereas Rachmaninoff's Vocalise, a slower cello and piano duet, was playing (Wang & Spence, 2015).

What remains unclear, though, on the basis of the research that has been published to date is whether expertise influences the crossmodal interaction between taste and sound. At first, it might seem as though wine experts would be “immune” to such perceptual biases because they are more experienced in the objective sensory evaluation of wine. However, that said, there is anecdotal evidence of winemakers and writers endorsing the effect of music on wine (Gray, 2007; Smith, 2010; see Spence & Wang, 2015b, for a review). On the other hand, if wine experts are more attuned to subtle differences in smell or taste (whether inborn or through training), then it is conceivable that they would be better able to pick out any subtle changes in the tasting experience, resulting from the way in which music changes the focus of their attention. Wine expertise could therefore act as a moderator on the way people perceive sound‐taste correspondences.

Training improves people's ability to discriminate flavors when tasting wine (Owen & Machamer, 1979). However, that is possibly because trained panelists and experts can adapt an analytical strategy that helps them to distinguish different components of wine flavors, when compared with untrained panelists (Arvisenet, Guichard, & Ballester, 2016). Moreover, wine experts use a different vocabulary when describing wines, using analytical terms, whereas nonexperts use holistic terms (Chollet & Valentin, 2000; Gawel, 1997). These observations might well lead to the suggestion that wine experts might be better at separating the influence of the music from their sensory evaluation of the wine itself. Furthermore, several neuroimaging studies involving wine have been conducted with the goal of pinpointing the influence of expertise on multisensory integration in wine evaluation. Sommeliers activate those brain regions that are involved in high‐level cognitive processes such as working memory and behavioral strategies when they taste wine—unlike novices who activate the primary gustatory cortex and emotional processing areas more (Castriota‐Scanderbeg et al., 2005). In a follow‐up study focused on the effect of expertise during the different phases of tasting (i.e., during vs. after tasting), Pazart, Comte, Magnin, Millot, and Moulin (2014) observed that wine experts activated those brain regions responsible for sensory integration immediately during the wine tasting phase, whereas for control participants they were only activated during the after tasting phase. This result implies that experts are able to analyze the sensory properties of wine more efficiently than untrained participants.

In terms of odors, expertise has been shown to increase sensitivity and discrimination (see Royet, Plailly, Saive, Veyrac, & Delon‐Martin, 2013; for a review), possibly giving rise to structural reorganization in olfactory brain regions (Delon‐Martin, Plailly, Fonlupt, Veyrac, & Royet, 2013). However, there seems to be no evidence that wine experts experience increased sensitivity when it comes to wine tasting. In fact, there seems to be no differences in sensitivity to odors in general—either those typically found in wine or otherwise (Brand & Brisson, 2012; Parr, Heatherbell, & White, 2002).

Taken together, then, the evidence that has been published to date suggests that while wine experts might have a different way of thinking about and describing wine, they are no more sensitive to detecting flavors. This is possibly because wine experts are trained to categorise and look for specific flavors or combination of flavors in a wine, and not to distinguish isolated flavors per se.

We conducted two studies on a population of highly trained wine specialists. First, we aimed to replicate the influence of specifically designed taste soundtracks on wine perception. These taste soundtracks were used in a music and Belgian beer study (Carvalho, Wang, Van Ee, & Spence, 2016), where sweet, sour, and bitter soundtracks were shown to influence the rated taste and strength of the beers. Second, we examined the effect of sound on more wine‐specific characteristics, such as length, balance, and body. In addition, it is also worth noting that both studies involved pairs of white wines. They were chosen to have the same color in order to control for the effect of color on wine evaluation, since most previous studies involving wine and music had used wines having distinctively different colors (e.g., Spence et al., 2013; Wang & Spence, 2015). The two studies were conducted one after another.

## 2 STUDY 1

### 2.1 Methods

#### 2.1.1 Participants

**Figure 1:**
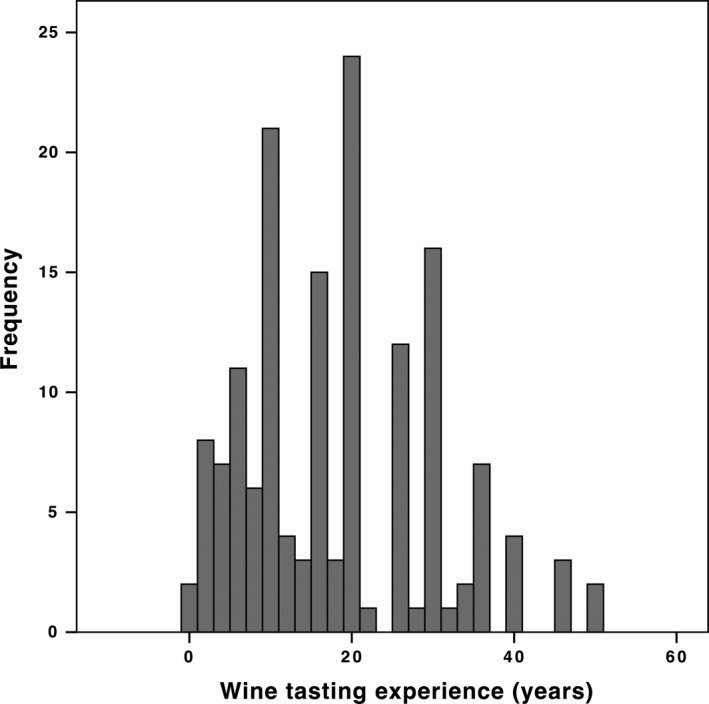
Histogram showing the distribution of participants’ wine tasting experience in terms of years

One hundred and fifty‐four participants (71 women, 79 men, four unspecified) between 20 and 75 years of age (*M* = 46.4, *SD* = 12.5) took part in the studies. One hundred and thirty‐eight of the participants were professionals working in wine‐related occupations, of which 42 works in education/research, 17 in journalism/wine writing, 13 in retail, three in restaurant service, and 63 in viniculture/viticulture. The participants were experienced wine tasters (see Figure 1 for a histogram), with a median of 16 years and an average of 18.1 years (*SD* = 11.5) of wine tasting experience (137/154 of the participants has over 5 years of wine tasting experience).

The participants gave their informed consent, and reported no impairments of hearing, smell, or taste. The participants were recruited from the International Cool Climate Wine Symposium 2016 who attended the Sensory Evaluation Seminar. The experiment was approved by the Central University Research Ethics Committee of Oxford University (MSD‐IDREC‐C1‐2014‐205).

#### 2.1.2 Auditory stimuli

Study one involved soundtracks designed to match with sweet and sour tastes. The two soundtracks have been shown to be reliably associated with sweet and sour tastes, respectively (Wang, Woods, & Spence, 2015) and were used recently in a study involving beer (Carvalho et al., 2016). The sweet soundtrack has bells, piano, and synthesizer, has consonant harmonies, and legato articulation. The sour soundtrack includes piccolo and clarinet, has dissonant harmony and staccato articulation. The soundtracks from both studies can be heard at https://soundcloud.com/janicewang09/sets/iccws-2016.

#### 2.1.3 Wines

Both Study 1 and Study 2 involved a pair of white wines. The wines were chosen to be different but similar, so that the participants would not necessarily assume the wines to be the same. Each pair had the same color to control for the effect of color on the evaluation of the wine.

Study 1 involved two white blends from England‐Bolney Lychgate White 2014 and 2015. The 2014 vintage is off‐dry with ripe aromas of lychee and passionfruit, with the sweetness balanced by crisp acidity. The wine consists of a blend of Muller‐Thurgau and Reichensteiner. The 2015 vintage is a fruity, zesty blend of Reichensteiner, Schonburger, and Wurzer grapes and has rich, ripe scents, and flavors of lychee and passion fruit which are balanced by a crisp, spicy acidity and refreshing elderflower notes. Both wines were water white in color.

#### 2.1.4 Procedure

The experiment took place at the International Cool Climate Wine Symposium. The participants sat at a table in front of a placemat with four glasses of wine on top (two glasses for Study 1 and two for Study 2), a cup of water, spittoons, and a paper questionnaire. The wines were identified on the placemats by random 3 digit codes. Before the actual study began, the participants specified their gender, age, and whether they worked in a wine‐related profession, and years of wine tasting experience.

The sweet soundtrack was played during the first trial, and the sour soundtrack during the second trial. For each trial, the participants were instructed to taste the wine once the soundtrack started. First, they were asked to list the top three flavors they perceived from the wine. Next, they rated the sweetness and acidity level of the wine on 0–10 scales (0 = no sweetness/acidity, 10 = very high sweetness/acidity) and how much they liked the wine (0 = not at all, 10 = very much). The participants were instructed to rinse their mouths out with water in between wines. Half the participants tasted the Lychgate White 2014 with the sweet soundtrack and the Lychgate White 2015 with the sour soundtrack, the other half tasted the wines in the reverse order.

The study lasted for approximately 10 min.

### 2.2 Results

**Figure 2:**
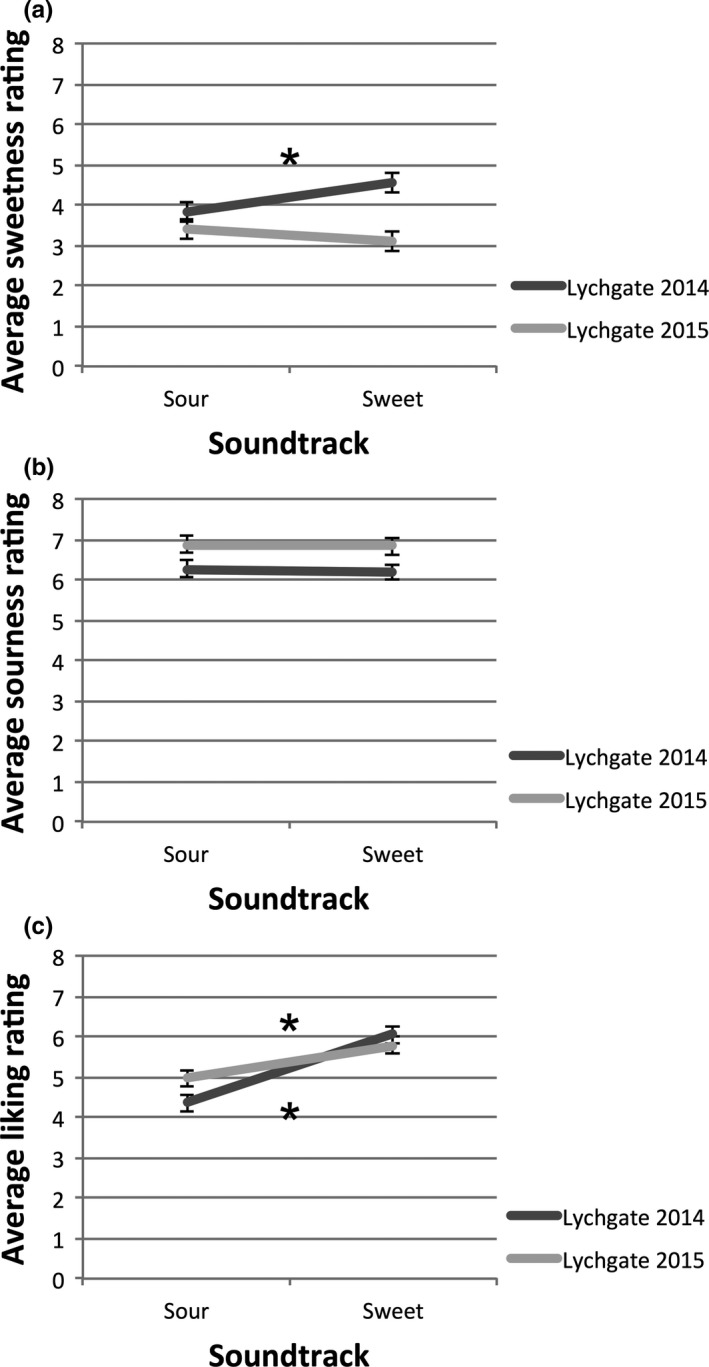
Mean values of sweetness (a), sourness (b), and liking (c) ratings in Study 1, with the sound conditions on the *x* axis and wine type shown as separate lines. Error bars indicate standard error. Asterisk “*”, indicates statistical significance at *p* < .05

Figure 2 shows the average values of sweetness, sourness, and liking ratings, grouped by music condition and wine type. A multivariate analysis of variance (MANOVA) test with wine (*x*2) and music (*x*2) as factors revealed a main effect of music (*F*_3,282_=10.99, *p* < .0005, Wilk's Lambda = 0.90), of wine (*F*_3,282_ = 5.96, *p* = .001, Wilk's Lambda = 0.94), and an interaction effect (*F*_3,282_ = 2.69, *p* = .047, Wilk's Lambda = 0.97). Further univariate ANOVAs revealed that music had a significant effect on wine liking (people liked the wine much better while listening to sweet music than to sour music, *F*_1,284_ = 32.97, *p* < .0005), but not on wine sweetness (*p* = .41) or sourness (*p* = .79). In terms of wine differences, the Lychgate 2014 was rated as significantly sweeter (*p* < .0005) and less sour (*p* = 0.004) than the Lychgate 2015, but there were no differences in how much the participants liked the two wines (*p* = .41). Most importantly, there was a significant interaction effect between music and wine type which was shown in ratings of sweetness and liking. For sweetness, the Lychgate 2014 was rated as sweeter during the sweet soundtrack than while listening to the sour soundtrack (*p* = .042), but not for the Lychgate 2015 (*p* = .38). For liking, the interaction effect comes from the fact that while both wines were liked equally during the sweet soundtrack, when listening to the sour soundtrack, participants preferred the more acidic Lychgate 2015 to the sweeter Lychgate 2014 (*p* = .043).

Moderation analysis was computed using the PROCESS macro (Hayes, 2013), using years of wine tasting as a moderator variable. No effect of moderation was found interacting between sound condition and ratings of sweetness (*p* = .18), sourness (*p* = .30), and wine liking (*p* = .80).

### 2.3 Discussion

Study 1 revealed that participants rated the wines as sweeter while listening to the sweet soundtrack compared to the sour soundtrack, as expected, but only for the sweeter of the two wines. This finding is in line with observations showing that a secondary sensory stimulus that is congruent with some flavor property can influence the evaluation of a food item only when the food item already has that flavor property. For instance, Shermer and Levitan (2014) reported that, as redness is associated with piquancy, increasing the intensity of red color only enhanced the piquancy of medium spicy—and not of mildly spicy—salsa. Similarly, a soundtrack composed to correspond with spiciness enhanced reported spiciness only for medium spicy and not mildly spicy salsas (see Wang, Keller, & Spence, 2017). In contrast, when it comes to the evaluation of wine liking, both wines were liked more when tasted while listening to the sweet soundtrack, regardless of the sweetness level of the wines. This seems to imply different mechanisms operating for taste evaluation (e.g., the soundtrack may have enhanced expectations) than for hedonic evaluation (e.g., the participants’ liking for the music may have transferred to their wine liking ratings).

Interestingly, the more acidic wine (Lychgate 2015) was liked more than the sweeter wine (Lychgate 2014) during the sour soundtrack, which suggests that music‐taste congruency could also have played a role in participants’ liking ratings. According to the theory of processing fluency (Labroo, Dhar, & Schwartz, 2008; Winkielman, Schwarz, Fazendeiro, & Reber, 2003), the better the match between the music and wine, the more easily participants could assess the tasting experience, and consequently, might find the wine more pleasant.

## 3 STUDY 2

Beyond the influence of soundtracks on basic taste attributes, the question remains as to whether sound can exert an effect other more complex features of wine. Given the high level of wine expertise of the participants, we examined the effect of sound on more wine‐specific characteristics, such as length (the duration of aftertaste), balance (how much the different components of wine are in harmony), and body (viscosity).

### 3.1 Methods

#### 3.1.1 Participants

The same participants who participated in Study 1 also took part in Study 2, which took place shortly after Study 1.

#### 3.1.2 Auditory stimuli

Study 2 involved two abstract soundscapes composed by Ben Houge, a researcher and sound designer specializing in aleatoric music composition (i.e., compositions that involve elements of random choice). The first soundtrack is sparsely textured and staccato, the second soundtrack is less sparse, with overlapping legato woodwind lines.

#### 3.1.3 Wines

Study 2 involved two Chardonnays from Ontario, Canada. Tawse Quarry Road Organic Chardonnay 2012 and 2013 Speck Family Reserve Chardonnay. Both wines were pale lemon in color. They presented fresh acidity, medium alcohol (13%), and had been aged in French oak barrels.

#### 3.1.4 Procedure

Study 2 took place five minutes after Study 1. The participants cleansed their palate with water in between studies.

The staccato soundtrack was played during the first trial, and the legato soundtrack was played during the second trial. For each trial, the participants were instructed to taste the wine once the soundtrack started. The wine attributes that the participants evaluated on 0–10 scales were: body (0 = very light, 10 = very full), balance (0 = not balanced, 10 = very balanced), length (0 = very short, 10 = very long, 10 +  seconds), wine liking (0 = not at all, 10 = very much), how well the music matches the wine (0 = not at all, 10 = very much), and music liking (0 = not at all, 10 = very much). The participants were instructed to rinse their mouths out with water in between wines. Half of the participants tasted the Quarry Road Chardonnay with the staccato soundtrack and the Speck Family Chardonnay with the legato soundtrack, and the other half tasted the wines in the reverse order.

The study lasted for approximately 10 min.

### 3.2 Results

**Figure 3:**
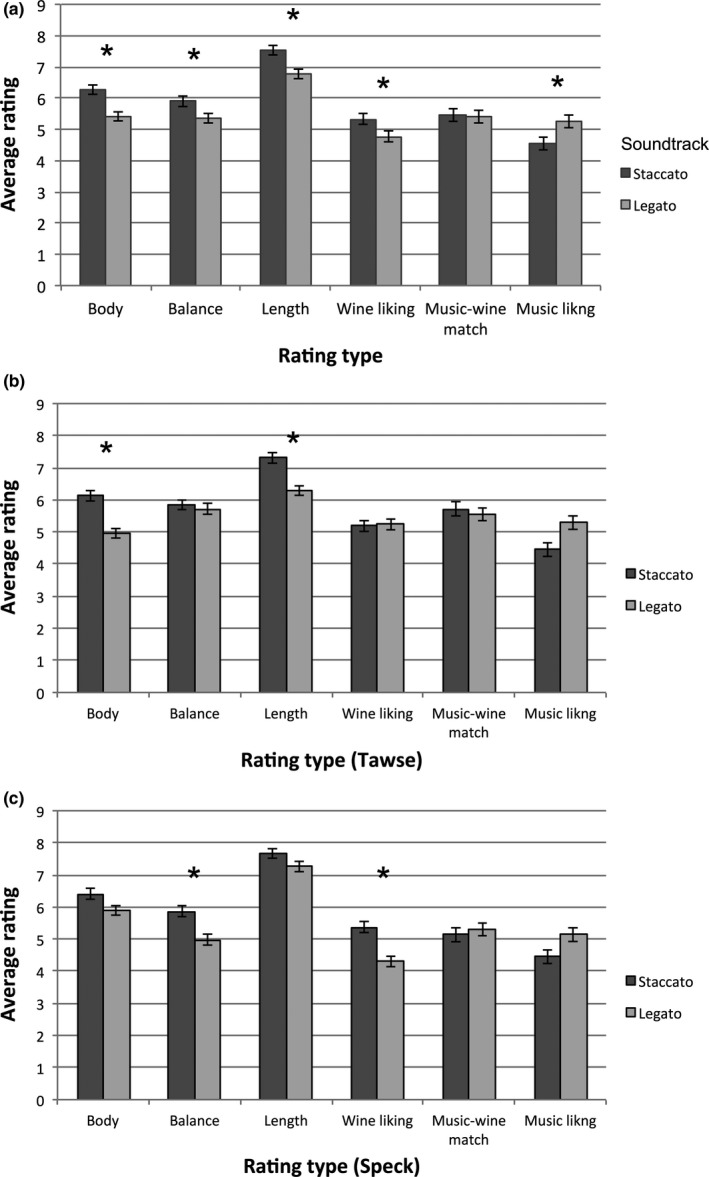
Mean values of wine body, balance, length, wine liking, music‐wine match, and music liking in Study 2, with the sound conditions shown in different colors. Results are averaged over both wines (a), and shown individually—Tawse Chardonnay (b), Speck Chardonnay (c). Error bars indicate standard errors. Asterisk ‘*’, indicates statistical significance at *p* < .05. All *p*‐values in post hoc comparison tests have been Bonferroni corrected

MANOVA with wine (*x*2) and music (*x*2) as factors again revealed a significant main effect of music (*F*_6,287_ = 6.49, *p* < .0005, Wilks’ Lambda = 0.88; see Figure 3), wine (*F*_6,287_ = 4.36, *p* < .0005, Wilks’ Lambda = .92), and an interaction effect (*F*_6,287_ = 3.05, *p* = .007, Wilks’ Lambda = .94). Further univariate ANOVAs showed that wines tasted while listening to the staccato soundtrack was rated as significantly fuller in body (*F*_1,292_ = 15.45, *p* < .0005), more balanced (*F*_1,292_ = 5.82, *p* = .016), having a longer finish (*F*_1,292_ = 11.66, *p* = .001), and liked more (*F*_1,292_ = 5.30, *p* = .022). That said, participants liked the staccato soundtrack significantly less than the legato soundtrack (*F*_1,292_ = 5.68, *p* = .018). In addition, the Speck winery Chardonnay was rated as having a fuller body (*F*_1,292_ = 6.07, *p* = .014) and a longer finish (*F*_1,292_ = 8.53, *p* = .004) than the Tawse Chardonnay. Finally, the interaction effect between music and wine can be observed in wine liking—the Speck Chardonnay was liked more while participants were listening to the staccato soundtrack as compared to the legato soundtrack (*p* = .001), but no such differences were observed for the Tawse Chardonnay (*p* = .96). All p‐values in post‐hoc pairwise comparisons have been Bonferroni corrected.

Moderation analysis was computed using the PROCESS macro (Hayes, 2013), using years of wine tasting as a moderator. No effect of moderation was found interacting between sound condition and ratings of body (*p* = .78), balance (*p* = .86), length (*p* = .66), wine liking (*p* = .80), music‐wine match (*p* = .35), and music liking (*p* = .80).

### 3.3 Discussion

The results of Study 2 revealed that the soundtracks influenced wine evaluation significantly in terms of body, balance, length, and wine liking. Somewhat surprisingly, listening to the relatively more disliked music (staccato soundtrack) was associated with fuller body, better balance, and longer length, features that are usually associated with greater wine enjoyment. While this seems counterintuitive at first, it might be possible that the sparseness of the staccato soundtrack provided a better contrast for the oaked Chardonnays and made them seem “fuller” in comparison.

Having a knowledgeable sample of participants meant more sophisticated wine‐related terms could be used in study. We show here that future studies could focus on the effect of music on more complex and nuanced food/drink characteristics, that is,, going beyond the basic tastes.

## 4 GENERAL DISCUSSION

In both studies, it can be seen that experienced wine tasters were also influenced by background music when it comes to wine evaluation. Moreover, years of wine tasting experience do not moderate the impact of music on wine evaluation. One might argue that the results observed here are in‐line with previous studies showing that wine experts exhibit no differences in olfactory sensitivity from novices (Brand & Brisson, 2012; Parr et al., 2002)—in other words, wine experts are not *unique* in their ability to perceive qualities in wines per se, but maybe in their ability to categories flavors and in what to expect (Hughson & Boakes, 2002). In this case, it is not surprising that wine experts can also be influenced by music, since music only seems to enhances/detracts certain flavors of the wine without creating new ones. Furthermore, it is worthwhile pointing out that this study involves a large number (154) of very experienced wine tasters (with an average of 18.1 years of experience), which makes this a unique study in the area of music‐wine correspondences.

That said, one limitation of the present studies is that we cannot rule out order effects as an explanation for the results we observed. Due to the public nature of the event, we could not counterbalance the music conditions across participants (even though we made sure to counterbalance the order in which the wines were tasted). In Study 1, the sweet soundtrack was played first, followed by the sour soundtrack. Therefore, it is possible that perception of pleasantness decreases with time (hence the wines tasted during the sweet soundtrack, which played first, was rated to be more pleasant than during the sour soundtrack). Similarly, in Study 2, because the staccato soundtrack was always played first, it's possible that the differences observed in terms of wine body, balance, length, and liking were due to presentation order. Further studies with a counterbalanced order of music presentation will be needed to examine this possible explanation.

In terms of practical applications, our results show that music‐wine experiences can be designed for those at various knowledge levels, beyond just novice drinkers.

## CONFLICT OF INTEREST

The authors declare that they have no conflict of interest.
